# Induction of *Catharanthus roseus* Secondary Metabolites When *Calotropis procera* Was Used as Bio-Stimulant

**DOI:** 10.3390/plants10081623

**Published:** 2021-08-06

**Authors:** Amany H. A. Abeed, Mohammed Ali, Esmat F. Ali, Ali Majrashi, Mamdouh A. Eissa

**Affiliations:** 1 Department of Botany & Microbiology, Faculty of Science, Assiut University, Assiut 71516, Egypt; dramany2015@aun.edu.eg; 2 Egyptian Deserts Gene Bank, Desert Research Center, Department of Genetic Resources, Cairo 11753, Egypt; mohammedalidrc@gmail.com; 3 Department of Biology, College of Science, Taif University, Taif 21944, Saudi Arabia; a.esmat@tu.edu.sa (E.F.A.); aa.majrashi@tu.edu.sa (A.M.); 4 Department of Soils and Water, Faculty of Agriculture, Assiut University, Assiut 71526, Egypt

**Keywords:** alkaloid, bio-stimulant, *Calotropis procera*, *Catharanthus roseus*, foliar spraying, phytochemical

## Abstract

Available information associated with *Calotropis procera* posted its phytotoxic effect as bio-herbicide scarce works studied its stimulatory/nutritive effect. A pot experiment was performed to assess the validity of using *Calotropis procera* (*C. procera*) leaves extract as a bio-stimulant for the growth and quality of a medicinal plant *Catharanthus roseus* (*C. roseus*) evaluated by some physio-biochemical indices. Different types of *C. procera* leaves extracts (CLEs) (methanolic, cold water and autoclaved water extracts) were delivered by two different modes of application. The results revealed that application of CLEs as irrigation or foliar spraying caused a stimulation effect on *C. roseus* plant. Root and shoot length, dry and fresh weight were significantly improved due to CLEs applications. *C. roseus* bioactive molecules such as anthocyanins, phenolics, flavonoids, alkaloids, ascorbic acid, reduced glutathione and α-tocopherol were abundance increased significantly with CLEs applications. Reactive oxygen species (ROS) decreased explaining the involvement of CLEs in induction of antioxidant enzymes catalase, ascorbate peroxidase, polyphenol oxidase, guaiacol peroxidase and glutathione-S-transferase for modifying cell oxidative status witnessed by lower lipid peroxidation that kept below the untreated plants’ baseline reflected the improvement of growth and quality rather than phytotoxic effect. The promotion of wholesome-promoting secondary metabolites by CLEs was closely correlated to elevated phenylalanineammonialyase activity. The comparable efficient effect induced by all treatments might be judged by the relation between *C. procera* phytochemicals and *C. roseus* metabolism (donor-receiver relation). It is concluded that application of CLEs can be a promising approach for improving the yield and quality of plants despite using polluting fertilizers. The current investigation may provide a matrix for coming studies to seek illustration of numerous plants’ response to *C. procera* extracts.

## 1. Introduction

Within the agricultural and horticultural industry, high yield and high-quality crops are of major commercial and economic importance. The demands for increased yield and improved quality can be met through improvements in crop genotypes by selection, breeding, and genetic engineering and by improvement of the crop growth environment through irrigation, fertilization, and the use of plant protection products. Chemical growth regulators are also one of the tools used to improve both crop growth and quality. However, the extensive and irrational use of these agro-chemicals as inevitable practice for plant production posed undesirable environmental impacts. An alternate strategy to reduce the dependence on synthetic growth regulators and encourage the use of botanical sources is considered safe for the environment and helps in achieving global food security sustainably and quality and being environmentally friendly [1]. Various plant extracts are being approved claiming to enhance crop yield and are marketed as bio-stimulants [2].

*Catharanthus roseus* (L.) (Apocynaceae) is a perennial tropical plant with pink or white flowers and formerly known as *Vinca rosea*. Being a medicinal plant and factory of various valuable secondary metabolites many attempts are seeking to increase them for agrochemical or pharmacological purposes [3,4]. Great efforts have been made to maximize production on a large scale. Using synthetic growth regulators/fertilizers is an integrated part of *Catharanthus* breeding for commercial production of economically significant secondary metabolites [3]. Applying botanical-source substances may serve as cheap, versatile and safe yielding alternative management and practice for important medicinal plants need to cope up with global increasing demands.

Plants are endowed with highly potent and versatile phytochemicals in their extracts. Phytochemicals, being secondary metabolites, promote root growth by improving moisture availability and temperature regulation, enhance mineralization of nutrients and improve their uptake [5]. They regulate seed germination, maturity, senescence, water relations, chlorophyll accumulation, photosynthesis, translocation of assimilates and quality, transpiration, leaf expansion, translocation and genetic encodings [4]. The positive role can be predicted at their lower concentrations. In this way, they can be introduced for growth promotion and can substitute for synthetic growth regulators. Recently, application of plants extracts as bio-stimulants to crops can be a cost-effective and efficient means to enhance growth and to improve crop productivity [1,5,6,7]. Although most of the research on the use of bio-stimulants has focused mainly on their benefits under different stress conditions, there have been also several reports on bio-stimulant-induced growth stimulation when they were applied to plants grown under normal growth conditions [8]. In fact, in dwindling agricultural lands, it should be a dominant goal of today’s plant researchers and breeders to explore the potential of new plant extracts as growth and quality improving substances of commercially valuable plants, including *Catharanthus roseus*, that could be promising cost-effective and substitute tool in achieving higher productivity and quality without compromising the environmental safety.

*Calotropis procera* (L.) (Asclepiadaceae), mudra, usher, apple of Sodom or giant milkweed, is a xerophytic perennial medicinal plant. It is native to tropical and subtropical Africa and Asia and widespread in the Middle East. It grows in Egypt, and it is the only species cited in that region. It is broadly distributed in Nile-Faiyum, Red Sea coastal region, and Gebel Elba and situated in the southeast corner of Egypt at Sinai proper and the Sudan frontier [9]. This highly invasive species can tolerate poor soils and adverse climatic conditions [10] and has reached the status of weed in many regions. Having high potential to invade pristine or economically important areas makes it of much concern, as it is also very difficult to eradicate, necessitates dealing with it to the maximum benefit. The plant received much concern from researchers due to its various biological effects owing to the extractable phytochemicals. Sizable research posted its phytotoxic effects, negative allelopathic potential and its role in weed control [11,12] while very limited works mentioned its nutritive and positive/stimulatory effect [13]. In the context, the active compounds from *C. procera* extract and their unique mode of action remain unknown due to the apparent multitude of potential molecular targets [12], performance overlapping and interaction between them [5,14], and their interference with the ecological factors regarding delivering method [15]. In our investigation, studying the impact of *C. procera* by using different types of extracts, ME, CWE and AWE (having different chemical natures) applied by two different modes, irrigation and foliar spraying (different contact surface, root and leaves), and identifying extracts composition may collectively present a clarification for the possibility to be used as bio-stimulant agent and mapping out how receiver plant could respond to them as well. Therefore, it seems valuable to assess and elucidate the physio-biochemical effects of *C. procera* phytochemicals that may catapult them as highly promising and environment-friendly natural substances and suitable for wider applications.

## 2. Material and Methods

### 2.1. Plant Materials and Tissue Collection

*C. procera* leaves were collected from the Agriculture Faculty Campus, University of Al-Azhar, Assiut, Egypt. *The Flora of Egypt* [16] was used for authentication of the plant. For phytochemical profile analysis four biological replicates from leaves were sampled from one-year-old *C. procera* plant. The method described by Naz and Bano [17] was used to prepare *C. procera* leaves extracts.

Three-month-old uniform *C. roseus* plants were collected in spring 2017 from the botanical garden of the Faculty of Agriculture, Assiut University. The plants were transferred into (30 cm in diameter and 35 cm in depth) containing 3000 gm air-dry soil (sand/clay 1:2 *v*/*v*) in rate of 5 plant/pot. The plants were kept in the greenhouse of the botanical garden of the Botany and Microbiology Department, Faculty of Science, Assiut University, Egypt (42″ and 28° 59′23″ E and latitude 25°45′06″ and 25°53′34″ N) to secure mild climatic conditions during experimentation.

### 2.2. Treatment

After one week of pre-culture, the transplants were randomly divided into irrigation and foliar spraying treatment groups. Each group consists of 12 pots (3 different extracts (methanolic extract (ME), cold water extract (CWE) and autoclaved water extracts (AWE)) × 4 replicates for each). The plants not delivered any extracts (either by irrigation or spraying) were considered as control. For irrigation treatment, each replicate was treated using extract by field capacity at the rate of 250 mL kg^−1^, soil with polyethylene bags to avoid soil treatment leaching. Additionally, for foliar spraying treatment, each replicate was sprayed by 250 mL of extract per pot and the soil surface was covered by polyethylene bag to avoid foliar treatment reaching soil. During the treatment, average temperature was between 21 °C and 27 °C and relative humidity varied between 65.4% and 70.5%, and plants were allowed to grow for one week. After one week of treatments, all the replicates were washed firstly thoroughly with tap water and then with distilled water to remove any extract surface deposit, rinsed in ultrapure water. Then the replicates were used for growth criteria measurements and physio-biochemical indices evaluation.

### 2.3. Isolation of Phytochemical Compounds from C. procera Leaves

The correct method to reduce technical variability throughout a sampling procedure is essential to stop cell metabolism and to avoid leaking of phytochemical metabolites during the various preparation steps before the actual phytochemical extraction [18,19]. Therefore, three biological replicates from the fresh leaves were immediately frozen on dry ice. In the laboratory, the frozen three biological replicates from fresh leaves samples were homogenized in cryogenic grinding for three cycles at one minute for each, after which the plant material (ca. 500 g, 280 g and 280 g) were directly soaked in 1000 mL from each of 90% methanol as a solvent, cold water and autoclaved water, respectively in Amber storage bottles, 2000 mL screw-top vials with silicone/PTFE septum lids (https://www.brecklandscientific.co.uk/aboutus.asp accessed on 1 January 2020) were used to limit dispersal of volatiles to the headspace then incubated with shaking at 37 °C and 200 rpm for 24 h [16,19]. Afterward, each one from ME, CWE and AWE were transferred and aliquoted into several 250 mL utofil^®^ PP centrifuge bottles with screw cap and centrifuged at 5000 rpm for 10 min at 4 °C to eliminate plant debris. The upper layers from the extract were transferred into new 1-L storage bottles. After that 3 mL from each extract was pipetted into three new glass tubes of 1.5 mL with lids to reduce a loss of volatiles, and each 1.5-mL glass tube contained 1 mL from the extract. Then all the glass tube 1.5 mL with lids were placed on the GC-MS auto-sampler for GC-MS run, or each tube was covered with paraflm after closed with screw-top vials with silicone/PTFE septum lids and stored at −20 °C until GC-MS analysis [18,19].

### 2.4. GC-MS Analysis of Phytochemical Compounds from C. procera Leaves

Trace GC-ISQ Quantum mass spectrometer system (Thermo Scientific, Austin, TX, USA) was used for phytochemical analysis. About 1 μL from each sample was injected into a GC-MS equipped with a TG–5MS column (30 m × 0.25 mm ID, 0.25 μm film thickness). Helium gas was used as a carrier at a constant flow of 1.0 mL min^−1^. The mass spectra were observed between 50–500 m/z. Temperature was initially started at 50 °C for 10 min, then increased at a rate of 5 °C min^−1^ to 250 °C, and isothermal was held at 300 °C for 2 min, and finally, the held isothermal for 10 min at 350 °C. NIST, Adams, Terpenoids and Volatile Organic Compounds libraries were used to identify the phytochemical constituents by comparing the recorded mass spectra for each compound with the data stored in the previous libraries. The relative percentage amount for each component was calculated using by Retention time index and comparing its average peak area with total peak areas. And each sample was represented by three replicates [18,19].

### 2.5. Determination of C. roseus Plant Growth Criteria Affected by C. procera Leaves Extracts under Irrigation and Foliar Spraying Treatments

After measuring the shoot and root length, the plants were separated into shoot and root parts for measuring fresh and dry weight. Shoots and roots length and fresh weight of harvested plants were determined immediately then oven-dried at 60 °C for 2 days to a constant weight to evaluate dry weight.

### 2.6. Determination of C. roseus Physio-Biochemical Indices Affected by C. procera Leaves Extracts under Irrigation and Foliar Spraying Treatments

#### 2.6.1. Photosynthetic Pigment Content

The component of leaf photosynthetic pigments; chlorophyll a, chlorophyll b, and carotenoids were quantified as mg g^−1^ FW adopting the method used by Abeed and Dawood [20].

#### 2.6.2. Primary Metabolites

Primary metabolites in terms of carbohydrate content, free amino acids content and soluble proteins content all expressed as mg g^−1^ DW were estimated according to the published methods of Abeed et al. [21] and Iqpal [22], respectively.

### 2.7. Stress Markers and Membrane Damage Trait

Oxidative stress was monitored by determined stress markers such as superoxide anion (µg g^−1^ FW, O_2_^•−^), hydroxyl radical (µmol g^−1^ FW, ^•^OH) and hydrogen peroxide (µmol g^−1^ FW, H_2_O_2_) using the method of Kamran et al. [23], Tripathi et al. [24] and Soobrattee et al. [25], respectively. Lipid peroxidation assessed as malondialdehyde (MDA) content expressed as µmol g^−1^ FW was quantified using the method of Madhava Rao and Sresty [26].

### 2.8. Determination of Secondary Metabolites, Non-Enzymatic and Enzymatic Antioxidant Capacities

#### 2.8.1. Secondary Metabolites and Non-Enzymatic Antioxidants

Anthocyanin pigments (mg g^−1^ FW), total flavonoid content (mg g^−1^ FW), alkaloids content (mg g^−1^ FW), total phenolic content (mg g^−1^ FW), ascorbic acid (µg g^−1^ FW), reduced glutathione (µg g^−1^ FW, GSH) and *α*-Tocopherol (µg g^−1^ FW) were determined as the methods of Krizek et al. [27], Khyade and Vaikos [28], Sreevidya and Mehrotra [29], Kofalvi and Nassuth [30], Colucci et al. [31], Anjum et al. [32] and Kivçak and Mert [33], respectively.

#### 2.8.2. Enzymatic Antioxidants

The enzymatic potential of leaves and root was detected by screening the activities of catalase (CAT; EC 1.11.1.6, u mg^−1^ protein g^−1^ FW min^−1^), ascorbate peroxidase (APX; EC 1.11.1.11, µmol mg^−1^ protein g^−1^ FW min^−1^), polyphenol oxidase (PPO/EC 1.10.3.1, u mg^−1^ protein g^−1^ FW min^−1^), guaiacol peroxidase (POD; EC 1.11.1.7, µmol mg^−1^ protein g^−1^ FW min^−1^), phenylalanine ammonialyase (PAL; EC 4.3.1.5, µmol mg^−1^ protein g^−1^ FW min^−1^) and glutathione-S-transferase (GST; EC 2.5.1.18, u mg^−1^ protein g^−1^ FW min^−1^) by following the methods adopted by Martinez et al. [34], Yaqoob et al. [35], Tatiana et al. [36], Parmeggiani et al. [37], Al-Zahrani et al. [38] and AbdElgawad et al. [39], respectively.

### 2.9. Statistical Analysis

A completely randomized design (CRD) was utilized for the pot experiments. Obtained data were expressed as means ± SE. SPSS 10.0 software program was used for performing the statistical analysis. Comparisons between control and treatments were assessed by one-way ANOVA using the least significant differences (LSD) test. Difference from control was counted significant at the probability levels of 0.05 or very significant at the probability levels of 0.01.

## 3. Results and Discussion

### 3.1. Identification of Phytochemical Components of C. procera Used Extracts

**Figure 1:**
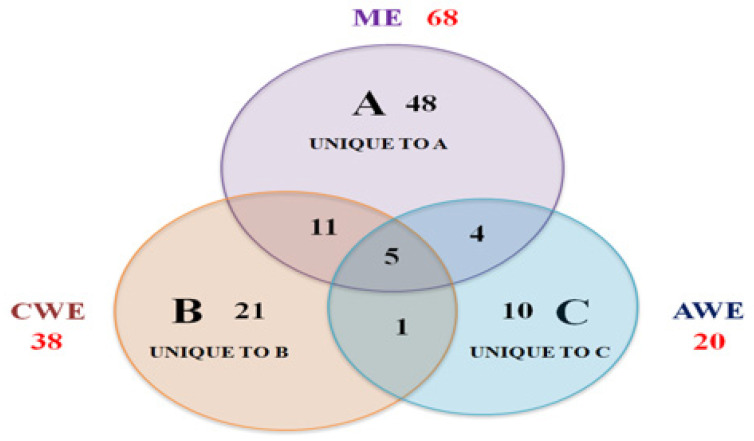
Three-way Venn Diagram to show the number of unique and common compounds in the three phytochemical extracts from *C. procera* leaves (**A**), methanolic extract (ME), (**B**) cold water extract (CWE) and (**C**) autoclaved water extract (AWE).

According to GC-MS analysis, 126 bioactive phytochemical compounds were identified in ME, CWE and AWE of *C. procera* leaves. The numbers of obtained bioactive phytochemical compounds in ME, CWE and AWE were 68, 38 and 20, respectively. The results of the qualitative and quantitative analyses of all phytochemicals in the three extracts are recorded in (Table 1 and Figure 1. The identified phytochemical compounds are listed based on the retention time, compound formula, compound molecular mass, CAS registry number and percentage of peak area (Table 1). In ME, guanosine was recorded as the major compound (15.57%), followed by 1-heptatriacotanol compound, neophytadiene compound and trans-phytol compound that registered as (4.57%), (4.53%) and (4.53%), respectively.

Regarding CWE, n-benzylidene-isopropylamine was recorded as the main compound (19.11%), followed by α-copaene compound (12.88%), followed by biocytin compound (9.51%) and 1,3-Dipalmitin, TMS derivative compound (6.61%). While phthalate (42.33%) was the main compound recorded in the AWE, followed by 2-Oleoylglycerol, 2TMS derivative compound (17.15%), followed by linoleic acid, 2,3-bis-(OTMS) propyl ester (α-glyceryl linoleate) compound (7.19%), followed by methyl 9,9-dideutero octadecanoate compound (5.83%) (Table 1). Moreover, each extract contained its unique and common phytochemical compounds that shared with others (Figure 1). For example, ME (A) had 48 unique compounds while shared with CWE and AWE by 11and 4 common compounds, respectively. Meanwhile, 5 common compounds were shared amongst all three leaf extracts. Furthermore, CWE (B) consisted of 21 unique compounds and one common compound shared with the AWE. On the other hand, AWE (C) contained 10 unique compounds (Figure 1). Thereby the variation existed in extracts composition and consequently different chemical nature is extract-type dependent.

### 3.2. Effect of C. procera Leaf Extracts on the Growth Criteria of C. roseus under Irrigation and Foliar Spraying Treatments

**Figure 2:**
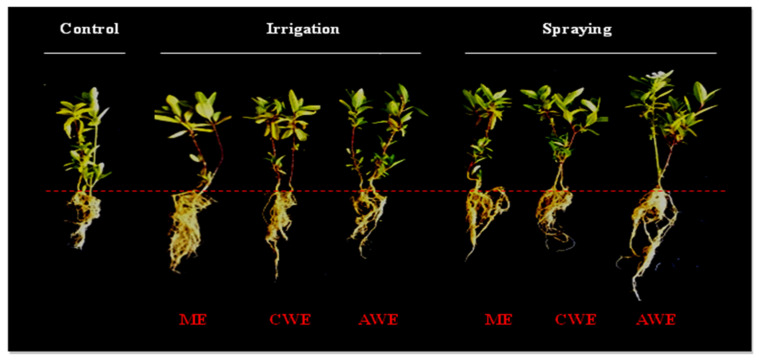
Illustration of growth pattern of *C. roseus* shoots and roots treated with three leaves extracts of *C. procera* (ME, CWE and AWE) applied by two different method (irrigation and foliar spraying) and control plants.

The shoot and root length of *C. roseus* increased significantly (*p* < 0.05 or *p* < 0.01) upon irrigation and spraying treatments. These increments ranged between 18–46% and 18–105% for shoot and root length, respectively, when compared with the control (Table 2 and Figure 2). The difference between the different types of extracts is significant in most cases (*p* ≤ 0.05) whereas their promoting effect was comparable regardless of the used application mode. On the other hand, highly significant (*p* < 0.01) growth promotion in terms of fresh and dry weight compared to control was registered by *C. procera* treatments which ranged between 25–160% and 55–85% for shoot and root fresh weight, respectively, when compared with the control. While dry weight increments ranged between 90–199% and 73–160% for shoot and root, respectively, as compared with the control. This effect upon fresh and dry weights was extract type and application mode independent as the values of fresh and dry weight statistically showed no significant differences between treatments (*p* > 0.05, bearing the same difference letters).

The obtained promotion effect on growth criteria is in agreement with the data recorded from studying the impacts of aqueous extracts of four medicinal plants (*Eclipta prostrate, Wood fordia fructicosa, Ageratum conyzoides* and *Cannabis sativa*) on the seed germination, seedling growth and biomass production of *Triticum aestivum* (wheat) and *Pisum sativum* (pea) [40]. Additionally, the obtained results are in line with Draz et al. [41] who demonstrated that the extracts from lantana, *Lantana camara*; henna, *Lawsonia inermis*; pomegranate, *Punica granatum*; acalypha, *Acalypha wilkesiana* and chinaberry, *Melia azedarach* significantly increased wheat yield components in terms of 1000-kernel and spike weight compared to the non-treated control. Moreover, Shabana et al. [42] and Nagwa and Iman [43] found that the foliar spraying of plant extracts (Brazilian pepper, pomegranate, neem, garlic, cactus, and eucalyptus) significantly increased wheat yield components, including spike weight and 1000-kernel weight. Naz and Bano [44] reported that *C. procera* leaves extract has growth-promoting effects on maize crop and this was attributed to the macro and micronutrients present in *C. procera* extracts easily absorbed by target plants that exhibit an influential role in the vital metabolism within the plant [45].

### 3.3. Effect of C. procera Leaves Extracts on Physio-Biochemical Indices of C. roseus under Irrigation and Foliar Spraying Treatments

#### 3.3.1. Photosynthetic Pigments Content

The results derived from growth-performance screening of *C. roseus* leaves and roots were depicted in Table 3 and Table 4. All *C. procera* extracts induce significant (*p* < 0.05) stimulant effect on photosynthetic pigments accumulation which recorded a considerable increment ranged between 60–179%, 71–156% and 32–158% for chlorophyll a, chlorophyll b and carotenoids, respectively, compared to control (Table 3). Statistically, in most cases, there is no significant difference between treatments. Stimulation effect was comparable between the different extracts of the two modes of application.

It is worthy to note that chlorophylls and carotenoids are ubiquitous and essential photosynthetic pigments, which are intricately linked with plant growth criteria in terms of shoot and root length, fresh and dry weight indicating enhancing carbon allocating process [46]. Draz et al. [41] found that plant extracts of *Acalypha wilkesiana*, *Melia azedarach*, *Lawsonia inermis*, *Lantana camara* and *Punica granatum* enhanced the total chlorophyll (a + b) content in wheat leaves.

#### 3.3.2. Primary Metabolites: Carbohydrates, Amino Acids and Proteins Content

Data illustrated in Table 3 revealed that three different types of extracts significantly (*p* < 0.05 or *p* < 0.01) intensified the primary metabolites production in terms of carbohydrates, amino acids and proteins in both leaves and root with percent increase ranged between 3–60%, 43–61% and 25–81% in leaves and 7–75%, 10–63% and 14–70% in root for carbohydrates, amino acids and proteins, respectively, in comparison to control. The overproduction of some primary metabolites (i.e., carbohydrates, amino acids and proteins) and consequently investment in *C. roseus* shoot and root biomass may go in line with the enhancing of chlorophyll content in the plant, presumably as a consequence of improved levels of photosynthetic pigments, leading to an increased photosynthetic rate have stimulated the source-to-sink transport of sugars thereby increasing carbohydrates content. The increasing in amino acids content goes parallel with the enhancing of proteins manufacturing indicating anabolism pathway under *C. procera* treatments.

These results were concomitant with Gamal et al. [47] who showed that the treatment with aqueous extracts of *Malva parviflora* L. and *Artemisia ludia* L. significantly increased the protein content, yield and growth of cowpea (*Vigna unguiculata* (L.) Walp.). Cheema et al. [6] and Gamalero and Glick [48] found that the phytochemicals can promote growth through optimum CO_2_ fixation upon normal conditions and they operate a positive role in physiological processes such as chlorophyll accumulate, photosynthesis, leaf expansion, translocation and genetic encodings, greater uptake and use efficiency of nitrogen resulting in a greater synthesis of protein.

#### 3.3.3. Superoxide Anion (O_2_^•−^), Hydroxyl Free Radical (^•^OH) and Hydrogen Peroxide (H_2_O_2_) Accumulation and Membrane Damage Trait (MDA)

Effect of *Calotropis* treatments on oxidative cell status of *C. roseus* was evaluated by determination of the stress markers (ROS), such as superoxide radical (O_2_^•−^), hydroxy radical (^•^OH), hydrogen peroxide (H_2_O_2_), besides ROS, oxidative burst to cellular membranes (membrane damage trait) assessed as lipid peroxidation level (malondialdehyde concentration, MDA) all of these were significantly (*p* < 0.05 or *p* < 0.01) and interestingly lowered than control by all applied treatments. Statistically (*p* ≤ 0.05) and in the most investigated cases of *Calotropis* treatments a comparable effect on MDA reduction was pronounced among all types of extracts submitting a comparable protective effect on *C. roseus* via reducing the accumulation of each O_2_^•−^, ^•^OH and H_2_O_2_ [49,50] consequently low MDA content in leaves and roots of *C. roseus***.** In the present study, CLEs-devoid control plants exhibited a substantial accumulation of ROS, including O_2_^•−^, ^•^OH and H_2_O_2_ which was accompanied by a concomitant increase in MDA content indicating that the control plants might have suffered from oxidative damage under field conditions. The reduction in ROS level was concomitant with enhancing of membrane integrity and stability evidenced by low MDA concentration that may be another main reason for growth improvement under *C. procera* treatments compared to control reflecting high optimal condition for efficient cellular metabolism and performance that can be ascribed to stabilization of cell redox status [49,51]. The improving mechanism displayed by *Calotropis* treatments thus was appraised by oxidative cell status maintenance ensuring normal cellular function and cell metabolism by *Calotropis* that was associated with enhancing in growth criteria, advocating bio-stimulant effect of *Calotropis* extracts upon *C. roseus*.

#### 3.3.4. Anthocyanin, Phenolics, Flavonoid, Alkaloids, Ascorbic Acid, Reduced Glutathione and α-Tocopherol Contents

*Catharanthus roseus* is one of the very extensively investigated medicinal plants due to its powerful antioxidants. It produces a wide spectrum of secondary metabolites viz., anthocyanins, phenolics and alkaloids. We further elucidated the beneficiary role of CLEs by quantifying the amount of non-enzymatic antioxidants. The protective impact of *C. procera* was explained by exacerbation of low molecular weight non-enzymatic antioxidants viz. ascorbic acid, reduced glutathione and α-tocopherol (which registered percent increase fluctuated between 19–40, 2–7 and 211–411, respectively) besides boosting of secondary metabolites pathway as manifested by elevated leaves anthocyanins, phenolics, flavonoids and alkaloids contents (Table 4) with percent increase ranging between 47–87, 6–31, 10–370 and 37–197, respectively, when compared to control. *C. procera* treatments significantly (*p* < 0.05 or *p* < 0.01) increased the antioxidant potential of *C. roseus* plant whatever extract type and application mode. These compounds play multiple functions concerning antioxidant properties and the ability to diminish free oxygen radicals conferring membrane stability, in turn, restricts the dispersal of free radicals and minimizes membrane lipids peroxidation. This may be partly accounted for upholding the low level of lipid peroxidation in plants subsequently higher membrane integrity compared to control plants [49,51]. That was a promising result to approve *C. procera* as a prospective protective agent. Moreover, the exacerbation of both primary and secondary metabolites production by *C. procera* extracts indicates the highly prompted and efficiently up-regulated carbon metabolism that may encourage the translocation of carbon to its sinks with an increment in the carbon pool ultimately allocated for secondary metabolism and thus greater production of secondary metabolites as well as growth and development [52,53], the matter that may catapult *C. procera* as an inducible agent. In the present work, the increase in the production of secondary metabolites: anthocyanin, phenolics and flavonoids could be witnessed by increasing in PAL activities submitting an up-regulation of plant secondary metabolite production jointed with enhanced PAL activity that is efficiently prompted by CLEs. This is mainly due to the fact that PAL is an enzyme, which synthesizes a precursor for various secondary metabolites production and a crucial regulation factor between primary and secondary metabolism [54]. On the other hand, the elevated amino acids content might be legalized the availability of the phenylalanine (Phe) as an elite substrate for PAL thus more Phe is available for secondary metabolites production [55].

#### 3.3.5. Enzymatic Antioxidants

The alternations in the capacities of enzymatic antioxidant of *C. roseus* leaves and roots under irrigation and spraying treatments with CLEs (ME, CWE and AWE) were showed in Table 4. All *C. procera* treatments significantly (*p* < 0.05 or *p* < 0.01) triggered the enzymatic antioxidants activities in *C. roseus* leaves and roots compared to control thus the stabilizing of cell oxidative status may be due to boosting of quenching hydrogen peroxide enzymes viz. CAT, APX, PPO, POD and GST. The highest increment in leaves enzymes activities was registered for both enzymes PPO and GST and amounted to 389% and 384%, respectively, when compared to control. In root CAT activity, the highest increment value that amounted as 217% with respect to control was recorded.

The increment of various ascorbate–glutathione pathway enzymes, POD and GST, could interpret the trapping of oxidative stress burst and healthiness of *C. roseus* plants [49]. The stimulation of PAL activity by various extracts was the main way for secondary metabolites enhancement as phenolics, flavonoids and anthocyanins as was reported by Ghasemzadeh et al. [53,54]. The activation of PPO could increase the plant defense responses against the pathogen attack. *C. procera* extracts and whatever the method of application efficiently induced the activity of PPO under natural conditions. Thus, further studies should be done to evaluate the role of *C. procera* in induction biochemical changes under biotic and a biotic stress.

Overall, The enhanced antioxidative capacity via increasing activities of CAT, APX, PPO, POD, GST and PAL as well as healthy promoting non-enzymatic antioxidants such as ascorbic acid, (AsA), glutathione (GSH), α-tocopherols, function together to maintain membrane integrity which advocated by lower lipid peroxidation indicating the potential role of phytochemicals in CLEs at the cellular level as modulators of gene expression, plant growth regulators and in signal transduction [54,56,57] indicating that CLEs mediated the promotion of antioxidant enzyme activities. These results were concomitant with several studies that confirmed that many plants extracts have defense enzymes stimulation properties. Goel and Paul [58] found that the PPO in tomato was stimulated by *Azadirachta indica* aqueous fruit extracts. Additionally, PAL, POD and PPO in cotton were activated by zimmu (*Allium cepa* L. and *Allium sativum* L.) [59], and PAL in barely prompted by aqueous extract of leaves of *Azadirachta indica* [60].

### 3.4. The Interplay between Phytochemical Compounds from C. procera Extracts and Metabolism of C. roseus Plant

Based on the statistical components accompanied with the data in Table 2, Table 3 and Table 4 where comparable enhancing effects were achieved by either two modes of application regarding growth criteria or antioxidants potential, it can be suggested that the behavior of phytochemicals of the tested extracts in the soil (rooting medium) was not the dominant factor determining the phytochemical performances but spraying (leave contact surface) treatments can produce comparable results as well. Besides, the nature of phytochemicals mostly differs according to the type of extract however the same result was also pronounced. Accordingly, the reaction between *C. procera* phytochemicals and of *C. roseus* metabolism herein may be received plant species dependence regardless of the chemical nature of phytochemical or contact surface (foliar spraying or rooting medium) explaining the superiority of *Catharanthus* in cellular up-regulate, utilize and successful management of *Calotropis* phytochemicals. Thereby, the current investigation could serve as a matrix for further studies to seek elucidation of several plants’ responses to *C. procera* extracts as a bio-stimulant agent.

In an attempt to reveal the mechanism of phytochemical components action and mapping out how receiver plant could respond to them, we try to know the various putatively biological activities of each component that has been identified in the used extracts by comparing against the Chemical Entities of Biological Interest (ChEBI) and Kyoto Encyclopedia of Genes and Genomes (KEGG) pathway databases [61].

#### 3.4.1. Guanosine (C00387)

Guanosine is a purine nucleoside, including guanine attached to a ribose (ribofuranose) ring through a β-N9-glycosidic bond, can be found in coffee plant, pollen of pines and clover [62]. It can be converted into guanosine monophosphate (GMP; C00144), cyclic guanosine monophosphate (cGMP; C00942), guanosine diphosphate (GDP; C00035), and guanosine triphosphate (GTP; C00044) by phosphorylation reaction. The previous forms have chief roles in biochemical processes such as proteins and nucleic acids synthesis, photosynthesis and intracellular signal transduction (cGMP), through different biochemical reactions such as (KEGG: R01227, R01228, R01677, R02145, R02147 and R02148) (KEGG: https://www.kegg.jp/dbget-bin/www_bget?C00387 (accessed on 1 January 2020)).

Moreover, the guanosine triphosphate (GTP; C00044) that produced from a small guanosine triphosphatases enzyme (small GTPases) are involved in nearly every aspect of cell biology such as, source of energy, an activator of substrates in metabolic reactions and it is one of the building blocks needed for the synthesis of RNA during the transcription process that may be a prerequisite for enzyme synthesis and eliciting in *C. roseus* [62]

#### 3.4.2. Biocytin (C05552)

Biocytinisan amide compound, a result from the vitamin biotin and the amino acid L-lysine, plays an important role in various pathways such as (biotin metabolism; KEGG: map00780, metabolic pathways; KEGG: map01100, vitamin digestion and absorption; KEGG: map04977) through different biochemical reactions such as (KEGG: R01077 and R04869) (KEGG: https://www.kegg.jp/dbget-bin/www_bget?C05552 (accessed on 1 January 2020)).

#### 3.4.3. Palmitic Acid (PA)

Palmitic acidor hexadecanoic acid group (C00249) such as (palmitoyl glycerol; 1,3-dipalmitin trimethylsilyl ether; palmitic acid, methyl ester and 1,2-dipalmitoyl-sn-glycerol-palmitin, 1,2-di-) [63] (KEGG: https://www.kegg.jp/dbget-bin/www_bget?C00249 (accessed on 1 January 2020)). Palmitic acid (PA) group plays important roles in biosynthesis of plant secondary metabolites and inducing PAL activity that the main reason herein that interpreted the augmentation of *C. roseus* secondary metabolites; KEGG: map01060, metabolic pathways; KEGG: map01100 and fatty acid metabolism; KEGG: map01212). They are associated with phosphatidyl inositols and has a role in the structure and functions of plant cell membranes [64].

#### 3.4.4. Phthalates (C01606)

Phthalatesor phthalate esters, are esters of phthalic anhydride and used in numerous applications especially as plasticizers to increase the durability, transparency, flexibility and longevity of plastics. Furthermore, in some edible plants, the exposure of phthalates has unequivocally affected the physiology, growth and development of edible plants [65,66] (KEGG: https://www.kegg.jp/dbget-bin/www_bget?C01606 (accessed on 1 January 2020)). Arpna and Rajinder [67], found that the barley seedlings treated with phthalate significantly increase the contents of proline, pigment, carbohydrate, soluble protein in barley seedlings shoots and roots. Additionally, CAT, SOD, POD, GR and APX activities were significantly increased in shoots and roots of barley seedlings after exposure by phthalate.

#### 3.4.5. Digitoxin (D00297)

Digitoxin is a phytosteroid and similar in structure to acetyldigitoxin (D01972), acetyldigoxin (D07556) and beta-Acetyldigoxin (D06881) (KEGG: https://www.kegg.jp/dbget-bin/www_bget?PATH:map07233 (accessed on 1 January 2020)). Phytosteroids in plants shoulder an important task as components of cell membranes and growth hormones serving as safeguarding factor for membrane function and stability that evidenced by low level of MDA in *C. roseus* by CLES supplementation [68]. Moreover, the digitoxin or other similar structure play roles in principal biological functions as signaling molecules, and can also modulate the activity of membrane-bound enzymes [69].

#### 3.4.6. Terpene and Terpenoid Groups

Terpene and terpenoid groups are a large and diverse class of organic compounds, produced by a variety of medicinal and aromatic plants [70]. Terpenes such as (+)-delta-cadinene (C06394), phytol (C05427), ascaridole (EMBL-EBI: CHEBI:2866) play important roles in various pathways such as (sesquiterpenoid and triterpenoid biosynthesis; KEGG: map00909, Biosynthesis of terpenoids and steroids; KEGG: map01062, metabolic pathways; KEGG: map01100, biosynthesis of secondary metabolites; KEGG: map01110, ubiquinone and other terpenoid-quinone biosynthesis; KEGG: map00130, terpenoid backbone biosynthesis; KEGG: map00900 and biosynthesis of plant secondary metabolites; KEGG: map01060) through different biochemical reactions such as (KEGG: R02311, R08371, R02063, R04795, R06284, R07500, R08756, R09067 and R12255) (KEGG: https://www.genome.jp/dbget-bin/www_bget?C06394; https://www.kegg.jp/dbget-bin/www_bget?C05427; EMBL-EBI: https://www.ebi.ac.uk/chebi/searchId.do?chebiId=CHEBI:2866 (accessed on 1 January 2020)). Moreover, certain terpenes cause plant development and growth hence considered primary rather than secondary metabolites [71].

#### 3.4.7. Carotenoids

Carotenoids are mainly terpenoids, and synthesized in various organisms (algae, bacteria, fungi and plants). Carotenoids such as astaxanthin (C08580), rhodopin (C19795), all-trans-beta-carotene (C02094), 4′-apo-beta,psi-caroten-4′-al (C19892) and alpha-carotene (C05433) play important roles in various pathways such as (carotenoid biosynthesis; KEGG: map00906, metabolic pathways; KEGG: map01100, biosynthesis of secondary metabolites; KEGG: map01110, retinol metabolism; KEGG: map00830, biosynthesis of various secondary metabolites—part 1; KEGG: map00999, biosynthesis of plant secondary metabolites; KEGG: map01060, biosynthesis of terpenoids and steroids; KEGG: map01062, biosynthesis of plant hormones; KEGG: map01070 and vitamin digestion and absorption; KEGG: map04977) through different biochemical reactions such as (KEGG: R07519, R07520, R07527, R07834, R09790, R07572, R07573, R08102, R00032, R03823, R03824, R05345, R07558, R07560, R07857, R08988, R09747, R10282, R10559, R12179, R09782 and R09783) (KEGG: https://www.kegg.jp/dbget-bin/www_bget?cpd:C08580; https://www.kegg.jp/dbget-bin/www_bget?cpd:C19795; https://www.kegg.jp/dbget-bin/www_bget?C19892; https://www.kegg.jp/dbget-bin/www_bget?C05433; https://www.kegg.jp/dbget-bin/www_bget?C02094 (accessed on 1 January 2020)).

Apocarotenoids also include many phytohormones with important functions in plant-environment interactions such as abscisic acid (ABA) and strigolactones (SL), and signaling molecules, such as β-cyclocitral that may help the acclimatization of *C. roseus* in its environment [72,73].

## 4. Conclusions

Exploring the potential of new plant extracts as growth and quality improving substances could be a promising, cost-effective and substitute tool in achieving higher productivity and quality without compromising environmental safety. *Calotropis procera* the broadly distributed ineradicable plant mostly posted as a toxic plant. Our study focused on exploring its inductive and protective properties that could be assessed by physio-biochemical indices. The results revealed that all CLEs triggered the biomass accumulation of *Catharanthus roseus* plant by the overproduction of primary metabolites, and upgrade plant quality via augmentation the production of secondary metabolites such as anthocyanin, phenolics, flavonoid and alkaloids. Furthermore, the improving mechanism that displayed *Calotropis* treatments was appraised by the oxidative status of *Catharanthus* cell. The enhanced antioxidative capacity via elicitation activities of CAT, APX, PPO, POD, GST and PAL as well as healthy promoting non-enzymatic antioxidants viz. ascorbic acid (AsA), glutathione (GSH), α-tocopherols function together to maintain cell oxidative status and were fostered from moderate to highly inducible rate to efficiently scavenge ROS this reflected on enhancement of membrane integrity witnessed by low MDA content. All this evidence directed us to approve *C. procera* as a bio-stimulant. Growth promotion imposed by *C. procera* leaves extracts reduces the fertilizer requirement in soil which limits the risk of overuse of fertilizer and outcoming fertilizer contamination in the environment. Application of *Calotropis* leaves extracts on medicinal plants could be utilized as a viable and sustainable green strategy for upgrading its medicinal property by augmenting secondary metabolites production.

## Figures and Tables

**Table 1:** GC-MS profile of different extracts from *Calotropis procera* leaves.

N	Compound Name	R.T	Formula	M.W	CAS	% Peak Area	Library
**Methanol Extract**							
1	Iron iodide complex I	4.56	C26H26FeIN4O4	641	NA	3.66	W
2	Palmitoyl glycerol	5.12	C19H38O4	330	542-44-9	0.55	W
3	2-Cyclohexylpiperidine	5.37	C11H21N	167	56528-77-9	0.41	M
4	Beta-d-Galactopyranoside, methyl 2,6-bis-*O*-(trimethylsilyl)-, cyclic b utylboronate	5.75	C17H37BO6Si2	404	56211-13-3	1.99	W
5	Octadecanedioic acid	6.89	C18H34O4	314	71-70-5	1.06	W
6	alpha-Carotene	18.01	C40H56	536.43	NA	0.7	M
7	2-octadecenoic acid, methyl ester	8.13	C19H36O2	296	14435-34-8	3.11	W
8	1,25-Dihydroxyvitamin D3, TMS derivative	8.49	C30H52O3Si	488	55759-94-9	1.13	W
9	Digitoxin	8.75	C41H64O13	764	71-63-6	0.53	R
10	Cholesta-5,7,9(11)-trien-3-ol acetate	9.47	C29H44O2	424	1255-91-0	0.36	M
11	Carbamic acid, N-methyl-N-[6-iodo-9-oxabicyclo [3.3.1]nonan-2-yl]-, ethyl ester	9.74	C12H20INO3	353	NA	0.81	M
12	7,8-Epoxylanostan-11-ol, 3-acetoxy-	10.81	C32H54O4	502	NA	0.77	M
13	beta-d-Galactopyranoside, methyl 2,6-bis-*O*-(trimethylsilyl)-, cyclic b utylboronate	11.64	C17H37BO6Si2	404	56211-13-3	0.82	W
14	Glucobrassicin	11.81	C16H20N2O9S2	448	4356-52-9	0.3	M
15	Geldaramycin	11.81	C29H40N2O9	560	30562-34-6	0.3	M
16	Sarreroside	12.14	C30H42O10	562	545-36-8	1.1	M
17	Indole-3-acetamide	12.47	C10H12N2O	176	50-67-9	0.26	W
18	2-linoleoylglycerol, 2TMS derivative	13.24	C27H54O4Si2	498	54284-46-7	0.4	W
19	Arachidonic acid, trimethylsilyl ester	13.66	C23H40O2Si	376	113516-18-0	0.96	M
20	Pseudosolasodine diacetate	14.09	C31H49NO4	499	NA	0.6	M
21	2-Trimethylsiloxy-6-hexadecenoic acid, methyl ester	14.41	C20H40O3Si,	356	NA	0.36	M
22	Lucenin-2	14.56	C27H30O16	610	29428-58-8	0.32	W
23	Alpha Methyl Mannoside	15.09	C15H28B2O6	326	54400-84-9	0.53	W
24	5,8,11-Eicosatriynoic acid, TMS derivative	15.51	C23H36O2Si,	372	NA	1.09	M
25	3′,4′,7-trimethylquercetin	15.79	C18H16O7	344	6068-80-0	0.22	W
26	Glyceryl 2-linoleateester, (Z,Z,Z)-	16.02	C27H52O4Si2	496	55521-23-8	2.09	W
27	à-d-Galactopyranose, 6-*O*-(trimethylsilyl)-, cyclic 1,2:3,4-bis(butylboronate)	16.67	C17H34B2O6Si	384	72347-47-8	0.27	M
28	Retinol	17.05	C20H30O	286	68-26-8	2.41	W
29	á-d-glucopyranoside, methyl 2,3-bis-o-(trimethylsilyl)-, cyclic methyl-boronate	17.52	C14H31BO6Si2	362	56211-07-5	1.71	W
31	Octadecanoic acid, 9,10-epoxy-18-(trimethylsiloxy)-, methyl ester, cis-	17.71	C22H44O4Si	400	22032-78-6	2.05	W
32	Bicyclo [4.4.0]dec-2-ene-4-ol,2-methyl-9-(prop-1-en-3-ol-2-yl)-	18.01	C15H24O2	236	NA	0.49	M
33	Guanosine	18.32	C10H13N5O5	283	118-00-3	15.57	W
34	Pentadecanoic acid	18.61	C15H30O2	242	1002-84-2	0.16	W
35	Neophytadiene	18.74	C20H38	278	504-96-1	4.53	M
36	Trans-Phytol	18.76	C20H40O	296	150-86-7	4.53	W
37	2-aminoethanethiol hydrogen sulfate (ester)	18.93	C2H7NO3S2	157	2937-53-3	0.27	W
38	[1,1′-bicyclopropyl]-2-octan oic acid, 2′-hexyl-, methyl ester	19.27	C21H38O2	322	56687-68-4	0.85	M
39	Ethanol, 2-(9-octadecenyloxy)-, (Z)-	19.54	C20H40O2	312	5353-25-3	2.42	W
40	9-Octadecenoic Acid, (2-Phenyl-1,3-Dioxolan-4-Yl)Methyl Ester, Cis-	19.83	C28H44O4	444	56599-45-2	0.68	M
41	5,8,11-Eicosatriynoic acid, methylester	20.01	C21H30O2	314	NA	1.26	M
42	Hexadecanoic acid, methylester	21.37	C17H34O2	270	112-39-0	3.22	W
43	tristrimethylsilyl ether derivative of 1,25-dihydroxy vitamin d2	22.28	C37H68O3Si3	644	NA	0.54	W
44	à-D-Glucofuranose, 6-*O*-(trimethylsilyl)-, cyclic 1,2:3,5-bis(butylboronate)	22.81	C17H34B2O6Si	384	72347-48-9	0.5	W
45	4-Hexyl-1-(7-methoxycarbonylheptyl) bicyclo [4.4.0]deca-2,5,7-triene	23.31	C25H40O2	372	NA	0.27	M
46	D-Mannitol,1,1′-*O*-1,16-hexadecanediylbis-	23.66	C28H58O12	586	119049-16-0	1.05	M
47	Ethyl iso-allocholate	24.01	C26H44O5	436	NA	1.39	M
48	11-Octadecenoic acid, methyl ester	24.22	C19H36O2	296	52380-33-3	4.43	M
49	Linoleic acid ethyl ester	24.44	C20H36O2	308	544-35-4	1.61	W
50	9,12,15-octadecatrienoic acid, methyl ester	24.84	C19H32O2	292	7361-80-0	2.64	W
51	9,12,15-Octadecatrienoic acid, 2-[(trimethylsilyl)oxy]-1-[[(trimethylsilyl) oxy]methyl]ethyl ester, (Z,Z,Z)-	25.23	C27H52O4Si2	496	55521-23-8	0.34	W
**Methanol Extract**							
52	Isochiapin B	26.55	C19H22O6	346	102607-34-1	0.39	W
53	Oxiraneoctanoic acid, 3-octyl-, cis-	26.84	C18H34O3	298	24560-98-3	0.25	R
54	2-palmitoyl glycerol	28	C19H38O4	330	542-44-9	1.05	W
55	2-hydroxy-3-[(9e)-9-octadecenoyloxy] propyl(9e)-9-octadecenoate	28.24	C39H72O5	620	2465-32-9	0.85	W
56	A-d-glucopyranoside, methyl 2-(acetylamino)-2-deoxy-3-*O*-(trimethylsilyl)-, cyclic methylboronate	28.63	C13H26BNO6Si,	331	54477-01-9	0.44	W
57	Mannopyranose, 1-*O*-(trimethylsilyl)-, 2,3:4,6-dibutaneboronate	29.28	C17H34B2O6Si	384	55712-55-5	0.24	M
58	Isokaempferide	31.27	C16H12O6	300	1592-70-7	0.6	W
59	9,12,15-Octadecatrienoicacid, 2,3-Bis[(Trimethylsilyl) Oxy]Propyl Ester, (Z,Z,Z)-	31.91	C27H52O4Si2	496	55521-22-7	0.35	W
60	1-Heptatriacotanol	32.83	C37H76O,	536	105794-58-9	4.57	M
61	9,12,15-Octadecatrienoic acid,2-phenyl-1,3-dioxan-5-yl ester	34.62	C28H40O4	440	56700-76-6	0.25	M
62	5,8,11-Eicosatriynoic acid, tert-butyldimethylsilyl ester	36.53	C26H42O2Si	414		0.27	M
63	Vitamin E	37.6	C29H50O2	430	59-02-9	2.39	M
64	A-d-galactopyranoside, methyl2,3-bis-o-(trimethylsilyl)-,cyclic methylboronate	39.19	C14H31BO6Si2	362	56211-08-6	0.53	W
65	3-(tetradecanoyloxy)-2-[(trimethylsilyl)oxy]propylmyristate	40.31	C34H68O5Si	584	NA	0.24	W
66	á-D-Galactopyranoside, methyl 2,6-bis-*O*-(trimethylsilyl)-, cyclic methylboronate	41.08	C14H31BO6Si2	362	56211-06-4	2.79	M
67	o-tetrakis(trimethylsilyl) 3,5-dihydroxy-2-(3-hydroxy-1-octenyl) cyclopentanehe ptanoate	41.72	C32H64O5Si4	640	NA	0.2	W
68	25-Norisopropyl-9,19-cyclolanostan-22-en-24-one,3-acetoxy-24 phenyl4,4,14 trimethyl-	42.57	C35H48O3	516	NA	0.14	M
**Cold Water Extract**							
1	1,3-Dipalmitin, TMS derivative	4.56	C38H76O5Si	640	53212-95-6	6.61	R
2	Palmitoyl glycerol	4.87	C19H38O4	330	542-44-9	1.24	W
3	Palmitic acid	5.12	C16H32O2	256	57-10-3	5.94	W
4	Ethyl iso-allocholate	6.89	C26H44O5	436	NA	2.4	W
5	1-Monooleoylglycerol trimethylsilyl ether	9.09	C27H56O4Si2	500	54284-48-9	0.66	W
6	Oxiraneundecanoic acid, 3-pentyl-, methyl ester, cis	9.29	C19H36O3	312	1041-25-4	1.06	W
7	Astaxanthin	9.88	C40H52O4	596	472-61-7	1.18	M
8	α-D-mannopyranoside	10.61	C15H28B2O6	326	54400-84-9	1.63	W
9	2H-Pyran,tetrahydro-2-(12-pentadecynyloxy)-	10.73	C20H36O2	308	56666-38-7	0.69	M
10	D-Mannitol,1,1′-*O*-1,16-hexadecanediylbis-	10.82	C28H58O12	586	119049-16-0	0.86	W
11	Biocytin	12.15	C16H28N4O4S	372	576-19-2	9.51	Nist_msms
12	Ascaridole	13.58	C10H16O2	168	135760-25-7	2.28	M
13	(+)-delta-Cadinene	14.09	C15H24	204		12.88	M
14	N-Benzylideneisopropylamine	14.37	C10H13N	147	6852-56-8	19.11	R
15	2-(3,4-dimethoxyphenyl)-3,5-dihydroxy-7-methoxy-4H-1-Benzopyran-4-one	15.1	C18H16O7	344	6068-80-0	1.08	W
16	3-Acetoxy-7,8-epoxylanostan-11-ol	16.18	C32H54O4	502	NA	0.37	W
17	5,8,11-Eicosatrienoic acid, (Z)-, TMS derivative	16.28	C23H42O2Si	378	NA	1.52	M
18	All-trans-beta-Carotene	18.01	C40H56	536.4	NA	1.19	M
19	1-Monooleoylglycerol, 2TMSderivative	18.45	C27H56O4Si2	500	54284-47-8	1.45	R
20	Tristrimethylsilyl ether derivative of 1,25-dihydroxy vitamin D2	18.74	C37H68O3Si3	644	NA	0.63	W
21	4′-Apo-beta,psi-caroten-4′-al	19.12	C35H46O	628	482.3549	0.63	W
22	5,8,11-Eicosatriynoic acid,tert-butyldimethylsilyl ester	19.55	C26H42O2Si	414	NA	1.98	M
23	Octadecanoic acid,9,10-epoxy-18-(trimethylsiloxy)-,methyl ester, cis-	19.61	C22H44O4Si	400	22032-78-6	0.66	M
24	10,12,14-Nonacosatriynoic acid	20.02	C29H46O2	426	NA	0.43	W
25	*9* *,* *12* *-* *Octadecadienoic acid* *(* *Z* *,* *Z)-, 2* *,* *3-bis[(trimethylsilyl* *)* *oxy* *]* *propyl ester*	20.55	C27H54O4Si2	498	54284-45-6	2	M
26	Rhodopin	20.69	C40H58O	554	105-92-0	1.37	W
27	Palmitic acid, methyl ester	21.37	C17H34O2	270	112-39-0	0.63	M
28	Methyl 2-*O*,3-*O*-bis(trimethylsilyl)-4-*O*,6-*O*-(methylboranediyl)-β-D-glucopyranoside	22.81	C14H31BO6Si2	362	56211-07-5	3.44	R
29	Methyl 2-*O*,3-*O*-bis(trimethylsilyl)-4-*O*,6-*O*-(methylboranediyl)-α-D-glucopyranoside	23.3	C14H31BO6Si2	362	54400-90-7	2.51	W
**Cold Water Extract**							
30	Glycodeoxycholic acid	23.59	C26H43NO5	449	360-65-6	0.67	M
31	à-D-Glucofuranose, 6-*O*-(trimethylsilyl)-, cyclic 1,2:3,5-bis(butylboronate)	23.65	C17H34B2O6Si	384	72347-48-9	0.49	W
32	1,25-Dihydroxyvitamin D3, TMS derivative	24.03	C30H52O3Si	488	55759-94-9	0.63	M
33	Oleic acid, methyl ester	24.21	C19H36O2	296	18654-84-7	4.25	W
34	7-Methyl-Z-tetradecen-1-ol acetate	24.45	C17H32O2	268		0.84	M
35	Glyceryl 2-linoleate	24.62	C27H52O4Si2	496	55521-23-8	1.08	W
36	Stigmasterol	26.27	C32H56OSi	484	14030-29-6	1.05	M
37	à-D-Galactopyranose, 6-*O*-(trimethyl-silyl)-, cyclic 1,2:3,4-bis(butylboronate)	27.67	C17H34B2O6Si	384	72347-47-8	0.77	M
38	Trilinolein	32.84	C57H98O6	878	537-40-6	1.28	M
**Autoclaved Water Extract**							
1	Linoleic acid, 2,3-bis-(OTMS) propyl ester (α-glyceryl linoleate)	4.56	C27H54O4Si2,	498	54284-45-6	7.19	W
2	5-(2-propenyl)-	15.8	C13H18O2	206	73685-60-6	1.97	W
3	2-Oleoylglycerol, 2TMS derivative	18.45	C27H56O4Si2	501	56554-42-8	17.15	M
4	methylboronate	20.21	C14H31BO6Si2,	362	56211-08-6	2.1	M
5	Palmitic acid, methyl ester	21.38	C17H34O2	270	112-39-0	3.18	R
6	5,8,11-Eicosatriynoic acid, tert-butyldimethylsilyl ester	23.57	C26H42O2Si	414	NA	1.54	M
7	Digitoxin	23.7	C41H64O13	764	71-63-6	1.42	M
8	Methyl 9,9-dideutero octadecanoate	24.22	C19H36D2O2	300	19905-64-7	5.83	W
9	Doconexent, TBDMS derivative	24.46	C28H46O2Si	442	NA	1.68	M
10	Phthalate	24.62	C8H6O4	166	88-99-3	42.33	M
11	Hexa-t-butylselenatrisiletane	31.28	C24H54SeSi3	506	93194-15-1	1.86	M
12	trimethylsilyl (13e)-9,11,15-tris[(trimet hylsilyl)oxy]prost-13-en-1-oate	34.79	C32H68O5Si4	644	55556-77-9	1.69	W
13	á-d-galactopyranoside, methyl 2,6-bis-o-(trimethylsilyl)-, cyclic methylboronate	34.87	C14H31BO6Si2	362	56211-06-4	0.75	M
14	spirosolan-3-ol, 28-acetyl-, acetate (ester), (3á,5à,22á,25s)-	35.26	C31H49NO4	499	1181-86-8	1.17	W
15	à-d-glucopyranoside, methyl 2,3-bis-o-(trimethylsilyl)-, cyclic methylboronate	35.36	C14H31BO6Si2	362	54400-90-7	1.97	W
16	5,8,11,14-Eicosatetraynoic acid, TBDMS derivative	36.3	C26H38O2Si	411	NA	1.68	M
17	à-D-Galactopyranose, 6-O-(trimethyl silyl)-, cyclic 1,2:3,4-bis(butylboronate)	36.35	C17H34B2O6Si	384	72347-47-8	0.88	M
18	o-tetrakis(trimethylsilyl)3,5-dihydroxy-2-(3-hydroxy-1-octenyl)-cyclopenta-neheptanoate	36.49	C32H64O5Si4	640	NA	1.49	W
19	bis(trimethylsilyl)derivative of3à,20à-dihydroxy-5à-pregnan-11-one	36.58	C27H50O3Si2	478	NA	1.49	M
20	Tristrimethylsilyl ether derivative of 1,25-dihydroxy vitamin D2	36.64	C37H68O3Si3	644	NA	1.62	W

**Table 2:** Effect of three types of *C. procera* leaf extracts (ME, CWE and AWE) applied by two different application modes (irrigation and foliar spraying) on growth criteria of *C. roseus*. Each value represents a mean value of four replicates ±SE. * and ** donate the difference significantly from the control at the probability levels of 0.05 and 0.01, respectively. Different letters within row (a, b, c) indicate significant differences (*p* ≤ 0.05) between the types of extracts of the two application modes irrigation and spraying.

Parameters		Irrigation			Foliar Spraying		
	Control	ME	CWE	AWE	ME	CWE	AWE
Shoot length (cm plant^−1^)	17.12 ± 0.11	25.01 ± 0.2 a **	20.91 ± 0.43 b **	20.33 ± 0.45 b **	20.13 ± 0.56 b **	23.09 ± 0.56 a **	25.01 ± 0.44 a **
Root length (cm plant^−1^)	8.44 ± 0.13	13.98 ± 0.3 a *	11.21 ± 0.22 b *	9.99 ± 0.31 b *	14.10 ± 0.34 a *	10.76 ± 0.3 1b *	17.34 ± 0.32 a **
Shoot fresh weight (g plant^−1^)	7.31 ± 0.22	10.98 ± 0.32 b **	9.13 ± 0.11 b **	10.21 ± 0.32 b **	13.63 ± 0.23 b **	18.21 ± 0.42 a **	19.01 ± 0.21 a **
Root fresh weight (g plant^−1^)	2.26 ± 0.18	3.75 ± 0.12 a **	3.85 ± 0.41 a **	3.56 ± 0.21 a **	3.98 ± 0.41 a **	3.85 ± 0.11 a **	4.18 ± 0.22 a **
Shoot dry weight (g plant^−1^)	1.46 ± 0.04	4.09 ± 0.23 a **	3.32 ± 0.23 a **	3.89 ± 0.14 a **	2.77 ± 0.14 b **	3.91 ± 0.22 a **	4.37 ± 0.11 a **
Root dry weight (g plant^−1^)	0.30 ± 0.02	0.78 ± 0.14 a **	0.52 ± 0.02 b **	0.59 ± 0.05 b **	0.59 ± 0.043 b **	0.54 ± 0.06 b **	0.78 ± 0.061 a **

ME: methanolic extract; CWE: cold water extracts; AWE: autoclaved extract. * Significantly different from control (*p* < 0.05) assessed by LSD test. ** Very significantly different from control (*p* < 0.01) assessed by LSD test.

**Table 3:** Effect of three types of *C. procera* leaves extracts (ME, CWE and AWE) applied by two different application modes (irrigation and foliar spraying) on physio-biochemical indices of *C. roseus*. Each value represents a mean value of four replicates ± SE. * and ** donate the difference significantly from the control at the probability levels of 0.05 and 0.01, respectively. Different letters (a, b, c) within row indicate significant differences (*p* ≤ 0.05) between the types of extracts of the two application modes irrigation and foliar spraying.

Physio-Biochemical Indices		Tissue Type		Irrigation			Foliar Spraying		
			Control	ME	CWE	AWE	ME	CWE	AWE
Pigments (mg g^−1^ FW)	Chlorophyll a	L	1.460 ± 0.43	2.320 ± 0.52 a *	2.59 ± 0.44 a *	4.080 ± 0.21 b **	3.210 ± 0.11 a *	2.260 ± 0.12 a *	3.970 ± 0.16 b *
	Chlorophyll b	L	0.303 ± 0.01	0.519 ± 0.02 a *	0.527 ± 0.04 a *	0.777 ± 0.05 b **	0.655 ± 0.07 b *	0.549 ± 0.03 a *	0.769 ± 0.05 b **
	Carotenoids	L	0.84 ± 0.11	1.812 ± 0.13 a *	1.497 ± 0.32 a	2.166 ± 0.021 b **	1.391 ± 0.11 a	1.112 ± 0.09 a	1.944 ± 0.09 b
Primary metabolites (mg g^−1^ DW)	Carbohydrates	L	15.916 ± 1.21	18.617 ± 1.55 a *	17.251 ± 0.92 a *	22.692 ± 1.87 b **	16.342 ± 1.34 a *	19.632 ± 1.32 a *	24.019 ± 1.88 b **
		R	9.524 ± 0.95	12.569 ± 1.12 a **	10.227 ± 0.66 a *	13.079 ± 1.01 a **	14.820 ± 0.99 b **	16.212 ± 1.32 b **	16.663 ± 0.93 b **
	Free amino acids	L	2.061 ± 0.34	3.131 ± 0.44 a *	2.948 ± 0.21 a *	3.312 ± 0.55 a **	3.210 ± 0.34 a *	3.017 ± 0.11 a *	3.219 ± 0.22 a *
		R	0.934 ± 0.03	1.214 ± 0.06 a *	1.023 ± 0.09 a *	1.352 ± 0.11 a *	1.334 ± 0.09 a *	1.109 ± 0.10 a *	1.521 ± 0.08 a *
	Proteins	L	12.352 ± 1.23	17.991 ± 1.01 b *	15.400 ± 0.91 a *	18.321 ± 0.81 b **	18.201 ± 0.80 b **	17.667 ± 0.72 b *	22.321 ± 0.92 c **
		R	7.965 ± 0.45	10.870 ± 0.65 a *	9.112 ± 0.44 a *	11.442 ± 0.89 a **	9. 14 ± 0.67 a *	9.632 ± 0.55 a *	13.521 ± 1.09 b **
Reactive oxygen species and membrane damage trait	O_2_^−^ (µg g^−1^ FW)	L	10.960 ± 0.55	9.101 ± 0.43 c *	8.674 ± 0.32 c *	7.001 ± 0.54 b **	6.985 ± 0.44 b **	6.642 ± 0.34 b **	5.488 ± 0.63 a **
		R	0.890 ± 0.01	0.690 ± 0.02 b **	0.720 ± 0.03 b *	0.760 ± 0.02 b *	0.710 ± 0.01 b *	0.520 ± 0.02 a **	0.420 ± 0.01 a **
	OH (µmol g^−1^ FW)	L	1.066 ± 0.22	0.508 ± 0.01 a **	0.899 ± 0.03 b *	1.034 ± 0.32 b *	0.902 ± 0.04 b *	0.878 ± 0.01 b *	0.876 ± 0.02 b *
		R	4.591 ± 0.09	1.235 ± 0.02 a **	3.816 ± 0.04 b *	3.720 ± 0.03 b *	2.517 ± 0.03 a *	3.545 ± 0.04 b *	3.644 ± 0.04 b *
	H_2_O_2_ (µmol g^−1^ FW)	L	8.626 ± 0.23	6.547 ± 0.43 b *	7.689 ± 0.11 c *	6.963 ± 0.23 b *	4.292 ± 0.11 a **	7.380 ± 0.41 c *	4.983 ± 0.21 a *
		R	2.369 ± 0.03	1.915 ± 0.01 b *	2.013 ± 0.02 b	2.054 ± 0.02 b	1.992 ± 0.03 b	1.563 ± 0.01 a *	1.201 ± 0.04 a *
	MDA (µmol g^−1^ FW)	L	34.783 ± 1.51	19.711 ± 0.91 a **	19.084 ± 0.87 a **	24.226 ± 1.23 a **	24.354 ± 1.45 a **	21.798 ± 1.33 a **	22.919 ± 1.45 a **
		R	72.466 ± 2.03	60.437 ± 1.35 b **	53.620 ± 1.22 a **	59.516 ± 2.22 a **	53.733 ± 1.65 a **	60.988 ± 2.06 b **	59.210 ± 0.99 a **

L: leaves, R: roots, ME: methanolic extract, CWE: cold water extract, AWE: autoclaved water extract, H_2_O_2_: hydrogen peroxide, O_2_^•-^: superoxide anion, ^•^OH: hydroxyl radical, MDA: malondialdehyde. * Significantly different from control (*p* < 0.05) assessed by LSD-test. ** Very significantly different from control (*p* < 0.01) assessed by LSD test.

**Table 4:** Effect of three types of *C. procera* leaves extracts (ME, CWE and AWE) applied by two different application modes (irrigation and foliar spraying) on physio-biochemical indices of *C. roseus*. Each value represents a mean value of four replicates ±SE. * and ** donate the difference significantly from the control at the probability levels of 0.05 and 0.01, respectively. Different letters (a, b, c) within row indicate significant differences (*p* ≤ 0.05) between the types of extracts of the two application modes irrigation and foliar spraying.

Physio-Biochemical Indices		Tissue Type		Irrigation			Foliar Spraying		
			Control	ME	CWE	AWE	ME	CWE	AWE
Secondary metabolites (mg g^−1^ FW)	**Anthocyanins**	L	0.613 ± 0.02	0.611 ± 0.03 a	0.678 ± 0.03 a	0.659 ± 0.04 a	1.019 ± 0.32 b *	0.899 ± 0.05 b *	1.147 ± 0.11 b *
	**Phenolics**	L	3.701 ± 0.13	4.073 ± 0.22 a	4.135 ± 0.14 a	4.022 ± 0.09 a	4.853 ± 0.11 a	3.938 ± 0.14 a	4.017 ± 0.21 a
		R	0.498 ± 0.02	1.076 ± 0.05 b *	0.926 ± 0.04 b *	1.222 ± 0.12 b *	0.535 ± 0.01 a	1.161 ± 0.3 b *	0.518 ± 0.02 a
	**Flavonoids**	L	54.758 ± 2.41	60.870 ± 3.11 a *	80.818 ± 2.17 a *	65.110 ± 3.01 a *	130.641 ± 4.12 b **	115.066 ± 1.33 b *	257.157 ± 6.03 c **
		R	3.698 ± 0.54	7.100 ± 0.32 b **	6.819 ± 0.21 b **	6.646 ± 0.11 b **	4.295 ± 0.43 a *	8.761 ± 0.42 c **	4.901 ± 0.12 a *
	**Alkaloids**	L	0.561 ± 0.01	1.666 ± 0.04 c *	0.767 ± 0.01 a *	0.912 ± 0.04 b*	1.743 ± 0.06 c *	0.974 ± 0.02 b *	0.942 ± 0.03 b *
Non-enzymatic antioxidants (µg g^−1^FW)	**Ascorbic acid**	L	0.293 ± 0.01	0.350 ± 0.02 a *	0.357 ± 0.02 a *	0.410 ± 0.01 a *	0.360 ± 0.03 a *	0.367 ± 0.01 a *	0.369 ± 0.02 a *
	**Reduced glutathione**	L	51.478 ± 2.11	55.068 ± 2.23 a *	52.662 ± 2.11 a	53.198 ± 3.10 a	55.056 ± 3.11 a *	55.167 ± 2.05 a *	51.309 ± 1.23 a
	**Tocopherol**	L	129.806 ± 5	662.786 ± 28 c **	403.471 ± 16 a **	552.740 ± 22 b **	493.175 ± 21 a **	467.679 ± 23 a **	591.472 ± 30 b **
Enzymatic antioxidants	**CAT** **(u mg^−1^ protein g^−1^ FW min^−1^)**	L	10.379 ± 0.54	13.215 ± 0.43 a *	16.354 ± 0.65 a *	16.412 ± 0.56 a **	14.650 ± 0.44 a *	15.321 ± 0.43 a *	16.219 ± 0.82 a *
		R	0.95 ± 0.05	2.60 ± 0.1 b **	2.63 ± 0.4 b **	3.01 ± 0.5 b **	1.01 ± 0.05 a *	1.11 ± 0.08 a *	1.02 ± 0.07 a *
	**APX** **(µmol mg^−1^ protein g^−1^FW min^−1^)**	L	33.641 ± 1.56	35.210 ± 2.01 a	34.960 ± 1.78 a	39.250 ± 2.33 b *	40.130 ± 2.62 b *	41.320 ± 2.16 b *	41.710 ± 2.09 b *
		R	10.632 ± 0.54	15.631 ± 0.65 b *	14.320 ± 0.43 b *	14.604 ± 0.55 b *	10.905 ± 0.43 a	11.231 ± 0.32 a	11.421 ± 0.43 a
	**PPO** **(u mg^−1^ protein g^−1^ FW min^−1^)**	L	4.365 ± 0.13	15.966 ± 0.97 a *	19.365 ± 0.99 b *	15.215 ± 0.77 a *	20.363 ± 0.99 b **	16.325 ± 0.65 a *	21.325 ± 0.91 b **
		R	8.251 ± 0.65	18.365 ± 0.96 b **	19.325 ± 0.87 b **	19.001 ± 0.87 b **	11.325 ± 0.55 a *	10.251 ± 0.56 a *	15.965 ± 0.76 ab **
	**POD** **(µmol mg^−1^ protein g^−1^ FW min^−1^)**	L	16.951 ± 0.91	19.000 ± 0.53 a *	19.642 ± 0.65 a *	22.632 ± 0.89 b **	24.012 ± 1.09 b **	26.352 ± 0.99 c **	28.112 ± 1.31 c **
		R	5.479 ± 0.44	8.931 ± 0.33 b *	6.952 ± 0.82 a *	7.024 ± 0.41 a **	7.950 ± 0.34 b **	8.651 ± 0.63 b **	9.167 ± 0.91 b **
	**PAL** **(µmol mg^−1^ protein g^−1^ FW min^−1^)**	L	15.203 ± 0.87	19.362 ± 0.91 a *	18.952 ± 0.82 a *	20.325 ± 0.77 a *	19.362 ± 0.92 b *	24.252 ± 1.01 b **	24.633 ± 1.03 b **
		R	18.025 ± 0.96	24.362 ± 1.22 b **	26.365 ± 1.54 b *	29.362 ± 1.88 b **	20.754 ± 1.54 a *	21.633 ± 1.20 a *	20.325 ± 1.11 a *
	**GST** **(u mg^−1^ protein g^−1^ FW min^−1^)**	L	6.325 ± 0.05	18.352 ± 0.62 a **	14.633 ± 0.71 a **	16.325 ± 0.56 a **	23.366 ± 0.93 b **	23.252 ± 0.82 b **	30.650 ± 1.20 c **
		R	9.363 ± 0.57	9.997 ± 0.43 a *	9.51 ± 0.36 a	10.633 ± 0.78 a *	10.633 ± 0.75 a *	16.325 ± 0.88 b **	20.325 ± 1.12 c **

L: leaves, R: roots, ME: methanolic extract, CWE: cold water extract, AWE: autoclaved water extract, CAT: catalase, APX: ascorbate peroxidase, PPO: polyphenol oxidase, POD: guaiacol peroxidase, PAL: phenylalanineammonialyase, GST: Glutathione-S-transferase. * significantly different from control (*p* < 0.05) assessed by LSD-test. ** very significantly different from control (*p* < 0.01) assessed by LSD test.

## Data Availability

All the data are in the published article.
